# C_2_ Resilient Photosynthesis: A Practical Option for Long-Term Stable Carbon Sinks?

**DOI:** 10.3390/biology15010005

**Published:** 2025-12-19

**Authors:** Junjie Zhu, Fengyue Chen

**Affiliations:** Guangxi Key Laboratory of Forest Ecology and Conservation, College of Forestry, Guangxi University, Nanning 530004, China; 2409301001@st.gxu.edu.cn

**Keywords:** C_2_ photosynthesis, carbon sink, resilience, climate change, photosynthetic evolution

## Abstract

In recent years, extreme climate events such as high temperatures and droughts have become increasingly frequent and intense, posing significant threats to the carbon sink stability of C_3_, C_4_, and CAM plants. As a result, identifying photosynthetic strategies that balance adaptability with resilience has emerged as a critical focus in carbon sink research. C_2_ photosynthesis offers a promising solution by recycling photorespiratory CO_2_ through the glycine shuttle between mesophyll cells (MCs) and bundle sheath cells (BSCs), thereby optimizing carbon concentration and recovery without additional ATP expenditure, thus minimizing carbon loss. This review provides a comprehensive analysis of the diversity, distribution, evolutionary status, and regulatory mechanisms of C_2_ photosynthesis, emphasizing its physiological and ecological resilience in carbon sequestration. In comparison to C_3_ and C_4_ pathways, C_2_ photosynthesis demonstrates distinct carbon sink resilience, positioning it as a vital strategy for addressing both current and future global climate challenges. The review also highlights existing gaps in C_2_ research, particularly in species identification, molecular mechanisms, and ecological studies, and recommends prioritizing these areas to fully harness its potential for enhancing climate resilience.

## Simple Summary

C_2_ photosynthesis enhances net CO_2_ assimilation by capturing, concentrating, and reassimilating CO_2_ released during photorespiration, all while minimizing the additional energy expenditure. This process significantly improves carbon uptake, particularly under stress conditions. This review provides an overview of the diversity, distribution, evolution, and environmental resilience of C_2_ plants, highlighting their potential to stabilize carbon assimilation in the face of climate variability. It also addresses critical research gaps, including the identification of additional C_2_ species and the need for a deeper understanding of their molecular and ecological mechanisms. The review advocates for a more focused research effort to fully exploit the potential of C_2_ photosynthesis in enhancing climate resilience.

## 1. Introduction

The carbon sink function refers to the process by which ecosystems capture atmospheric CO_2_ and convert it into organic carbon through photosynthesis, thereby storing it long-term [1]. As the primary metabolic pathway for carbon fixation, plant photosynthetic pathways directly influence the rate, magnitude, and stability of carbon sequestration by regulating key enzyme activities, optimizing CO_2_ supply efficiency, and minimizing photorespiratory losses [2]. However, global climate change is significantly altering the carbon absorption patterns of terrestrial ecosystems, imposing complex impacts on the carbon sequestration capacity of plants with different photosynthetic pathways. In this context, climate projections indicate that future growing seasons will generally be hotter and drier, characterized by more uneven precipitation and increased climate variability. This issue is especially pressing in tropical regions, where extreme weather events—such as droughts followed by floods—frequently disrupt plant growth, severely undermining carbon sequestration capacity. Furthermore, atmospheric CO_2_ concentrations are expected to exceed 500 μmol/mol by 2050, which will impact plants with different photosynthetic pathways in varying ways [1].

For C_3_ plants, high-CO_2_ environments confer theoretical advantages: elevated CO_2_ concentrations increase Rubisco’s affinity for CO_2_, reduce photorespiration, and enhance overall carbon fixation, providing growth advantages under normal climatic conditions [3]. However, these benefits are largely negated under high-temperature and drought stress. High temperatures increase Rubisco’s oxygenase activity, reinstating photorespiratory activity, while drought stress induces stomatal closure, thereby reducing CO_2_ availability to the plant. These combined factors exacerbate photorespiration, and elevated CO_2_ concentrations can perturb the carbon-to-nitrogen (C/N) ratio and trigger source-sink imbalances, further reducing the carbon sequestration efficiency of C_3_ plants [4]. In contrast, C_4_ plants intrinsically mitigate photorespiratory losses through their unique CO_2_ concentration mechanism, augmenting photosynthetic efficiency and water use efficiency (WUE) under high-temperature and high-irradiance conditions. However, in cooler regions with abundant rainfall, their photosynthetic carbon sequestration capacity is significantly constrained, limiting their potential [5]. While CAM plants can maintain a degree of carbon sink functionality in arid environments by fixing CO_2_ at night and minimizing water loss, their low productivity and narrow distribution make them ill-suited for large-scale carbon sequestration applications [6].

Given the environmental fluctuations induced by global climate change, plants relying on a single photosynthetic pathway face inherent limitations in maintaining stable carbon sinks. Therefore, identifying photosynthetic strategies that can adapt to diverse environmental stresses while sustaining carbon sink functions has become a key focus in carbon sequestration research. Fortunately, a distinctive photosynthetic pathway has garnered growing attention in recent years. In the 1970s, Kennedy and Laetsch first identified the C_2_ photosynthetic pathway, classifying it as a C_3_–C_4_ intermediate [7]. This perspective prevailed for nearly four decades until Sage’s 2012 study demonstrated that C_2_ photosynthesis represents a transitional strategy in the evolutionary shift from C_3_ to C_4_ photosynthesis [8]. C_2_ plants concentrate CO_2_ through photorespiratory glycine shuttling: glycine is synthesized in mesophyll cells (MCs), transported to BSCs for decarboxylation, and the released CO_2_ is re-fixed by Rubisco in the BSCs [9]. This “glycine shuttle” or “photorespiratory CO_2_ pump” reduces the CO_2_ compensation point, mitigates photorespiratory carbon losses, and improves carbon assimilation efficiency. Notably, C_2_ plants exhibit remarkable metabolic plasticity, with the ability to switch between C_3_ and C_3_–C_4_ photosynthetic modes depending on environmental conditions. Some species even exhibit weak C_4_ traits [10,11,12]. This flexibility mitigates photorespiration during stomatal closure and aids C_2_ plants in adapting to saline soils, which can exacerbate photorespiration, endowing C_2_ photosynthesis with distinct adaptive advantages in flood-prone areas associated with soil salinization. Studies have shown that C_2_ plants not only recover photorespiratory CO_2_ efficiently but also enhance WUE in low CO_2_ environments, where photorespiration tends to increase. Their improved adaptability to climate change and tolerance to abiotic stresses [13] position them as promising candidates for resilient carbon sinks in the face of global climate change. As research on C_2_ plants progresses, significant advances have been made in understanding their species diversity, geographical distribution, phylogenetic relationships, structural characteristics, and physiological and molecular mechanisms. Comparative studies with C_3_ and C_4_ plants have elucidated the carbon fixation resilience of C_2_ plants and their potential as carbon sinks. However, challenges remain, including the complexity of C_2_ plant identification, an incomplete understanding of their molecular mechanisms compared to C_3_ and C_4_ plants, and unresolved issues related to the genetic stability of engineered C_2_ plants.

This paper provides a systematic review of the diversity, distribution patterns, evolutionary status, and physiological and molecular mechanisms of C_2_ photosynthesis. By comparing it with C_3_ and C_4_ pathways, it further explores C_2_ photosynthesis’ performance under both normal and stressed conditions, emphasizes its resilience in maintaining global carbon sink stability, and lays the groundwork for future research and development in this area.

## 2. Diversity, Distribution, and Evolutionary Status of C_2_ Plants

Sage and Khoshravesh proposed the concept of passive CO_2_ concentration mechanisms (pCCMs), in which plants do not expend additional ATP but instead “trap” CO_2_ released during photorespiration or respiration in a localized region around Rubisco [14]. A diffusion barrier between this region and the leaf’s outer airspaces promotes CO_2_ enrichment in this localized microenvironment. They established four criteria for identifying passive carbon concentration mechanism-dependent carbon concentration mechanisms (CCMDE): No additional ATP or reducing power is consumed. Local CO_2_ concentrations exceed those achievable through atmospheric diffusion. CO_2_ enrichment is facilitated by metabolic CO_2_ release or structural adaptations. A diffusion barrier exists between the high-CO_2_ region and the leaf’s outer airspaces. Based on these criteria, the C_2_ photosynthetic pathway is considered one of the most efficient forms of pCCMs in higher plants [14], a view that is widely accepted within the scientific community. Khoshravesh et al. further advanced the identification of C_2_ plants by developing reliable methods, including the detection of high-activity glycine decarboxylase (GDC) in BSCs, immunohistochemical analyses, ultrastructural observations of leaf tissues, and measurement of CO_2_ compensation points [15]. However, the practical implementation of these techniques remains laborious, underscoring the need for more efficient and accessible identification protocols in future research. To date, the C_2_ photosynthetic pathway has been documented in over 70 species across 13 families, including 4 monocot and 9 eudicot groups. Prominent plant families with abundant C_2_ species include Poaceae, Acanthaceae, Asteraceae, Amaranthaceae, Boraginaceae, Brassicaceae, and Portulacaceae (Table 1). C_2_ plants display substantial intraspecific and intraindividual photosynthetic diversity and plasticity [11], suggesting that additional C_2_ species are likely to be discovered in the foreseeable future.

Geographically, C_2_ plants are distributed across all continents except Antarctica, with greater representation in the Southern and Western Hemispheres. They are particularly abundant in regions such as Australia, the Americas, Western Europe’s Atlantic coast, and South Africa. Furthermore, C_2_ plants show considerable ecological and geographical diversity, encompassing both widespread and endemic species [47,48].

There are three primary hypotheses concerning the evolutionary status of C_2_ plants:

1. The Evolutionary “Bridge” Hypothesis: This hypothesis posits that C_2_ plants function as an evolutionary intermediate, mediating the evolutionary transition from C_3_ to C_4_ photosynthesis, thus laying the groundwork for the eventual evolution of C_4_ plants. Two supporting subtheories are proposed: the Nitrogen Hypothesis, which suggests that reduced photorespiration in C_3_ plants (through glycine shuttling, which releases ammonium into the bundle sheath cells (BSCs)) perturbs foliar nitrogen metabolism. To restore this balance, C_2_ photosynthesis would evolve into C_4_ photosynthesis, which is more nitrogen-use efficient [49,50]. The Environmental Hypothesis argues that C_2_ photosynthesis enhances carbon assimilation efficiency under high-temperature conditions, thereby enabling plant lineages to colonize warmer habitats than those occupied by their C_3_ relatives. These environments amplify photorespiratory losses, thereby imposing selective pressure on C_2_ plants and accelerating their evolutionary transition to C_4_ photosynthesis [26,51].
2. The Stable Photosynthetic Type Hypothesis: According to this hypothesis, C_2_ photosynthesis represents a stable photosynthetic strategy that is evolutionarily parallel to C_3_ and C_4_ pathways, and does not necessarily evolve into C_4_ photosynthesis. Supporting evidence includes: (1) anatomical constraints on metabolite exchange and cooler climates that inhibit the further evolution of C_2_ photosynthesis into C_4_, (2) the physiological adequacy of C_2_ photosynthesis for plant survival in certain environments, and (3) the persistence of C_2_ photosynthesis within specific lineages (e.g., Portulaca species) for millions of years without transitioning to C_4_ photosynthesis [52]. Furthermore, in Chenopodiaceae, C_2_-type species exhibit distinct upregulation of transcription factors, further suggesting that C_2_ represents an evolutionarily stable state within these taxa [15].
3. The Hybrid Origin Hypothesis: This theory proposes that C_2_ photosynthesis originates from interspecific hybridization events between C_3_ and C_4_ lineages. For example, the C_2_-type *Salsola divaricata* complex (Amaranthaceae) results from hybridization between C_3_ and C_4_ ancestors, enabling the species to adapt to a wider range of climatic conditions [23]. Similar hybrid-origin phenomena have also been observed in species such as *Diplotaxis* and *Homolepis isocalycia* [53,54,55].

## 3. Brief Overview of the Mechanisms of C_2_ Photosynthesis

C_2_ photosynthesis functions primarily as a photorespiratory carbon pump, recycling and reutilizing CO_2_ released during photorespiration through a series of anatomical, physiological, biochemical, and molecular processes.

### 3.1. Leaf Structural Adaptations in C_2_ Photosynthesis

**Figure 1:**
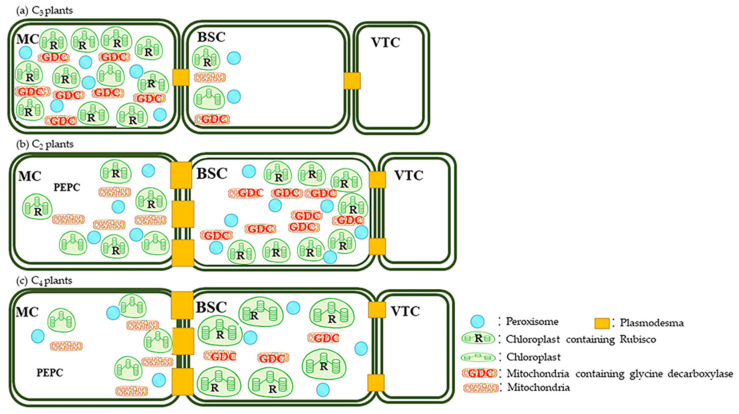
A schematic diagram illustrating the primary cellular architecture, key organelles, and core photosynthetic enzymes in the leaves of C3 (**a**), C2 (**b**), and C4 (**c**) plants. Detailed comparative analyses of leaf anatomical structures among the three plant types are provided in Table S1. Abbreviations: MC: Mesophyll cell, BSC: Bundle sheath cell, VTC: Vascular tissue cell.

Anatomically, C_2_ plants (both monocots and eudicots) possess Kranz-like leaf anatomical architectures in contrast to C_3_ plants. Notably, the ratio of BSC area to MC area is increased, and organelles (notably mitochondria and chloroplasts) are more densely packed, often concentrated near the BS cell walls (Figure 1).

This anatomical configuration provides the structural foundation for CO_2_ concentration: CO_2_ released during photorespiration accumulates in the BSCs, thereby enhancing Rubisco’s carboxylation efficiency. Furthermore, the density of plasmodesmata at the interface between BSCs and parenchyma cells is increased, facilitating metabolite transport. These anatomical features are integral to the efficient concentration of CO_2_ within the BSCs—a key component of the C_2_ photosynthetic pathway [56,57]. For a detailed comparison of leaf traits among C_2_, C_3_, and C_4_ plants, refer to Supplementary Materials Table S1.

### 3.2. Core Physiological and Biochemical Mechanisms of C_2_ Photosynthesis

A key characteristic of C_2_ photosynthesis is the specific localization of glycine decarboxylase within BSCs. In plants that utilize C_2_ photosynthesis, glycine decarboxylase (GDC) is mainly found in the mitochondria of BSCs, with significantly reduced activity in MCs [58,59]. This enzyme catalyzes the decarboxylation of two glycine molecules, producing one serine molecule while releasing one molecule of carbon dioxide and ammonia. GDC consists of four subunits: the P-protein, which contains a pyridoxal phosphate cofactor; the T-protein, which contains tetrahydrofolate; the H-protein, which contains a lipoamide cofactor; and the L-protein. Among these subunits, the P-protein functions as the key catalytic site. The cell-specific distribution of this enzyme enables the transport of glycine—produced through photorespiration—between MCs and BSCs. Specifically, glycine is synthesized in MCs, then transported to BSCs where decarboxylation occurs. The carbon dioxide released during this process is subsequently re-fixed by the enzyme Rubisco in BSCs. This mechanism reduces carbon loss associated with photorespiration and improves overall carbon assimilation efficiency. Research has shown that the carbon dioxide compensation point of C_2_ photosynthetic plants typically ranges from 10 to 40 micromoles per mole, which is considerably lower than that of C_3_ photosynthetic plants but higher than that of C_4_ photosynthetic plants (less than 10 micromoles per mole). Short-term ^14^CO_2_ labeling experiments and model analyses have revealed that in *Flaveria pubescens*—a plant that employs C_2_ photosynthesis—the in vivo ratio of carboxylation to oxygenation is more than three times higher than that of C_3_ photosynthetic plants [60]. This finding provides evidence that the photorespiratory carbon pump effectively increases the concentration of carbon dioxide in BSCs, a critical factor in enhancing the carbon fixation efficiency of C_2_ photosynthetic plants.

### 3.3. Key Molecular Mechanisms of C_2_ Photosynthesis

At the molecular level, the establishment of C_2_ photosynthesis involves extensive regulation of gene expression and reprogramming of metabolic networks. Several key molecular mechanisms underpin this process, including the re-localization of GDC, transcription factor regulation, and metabolite accumulation. The specific localization of GDC in BSCs is a central molecular event in the evolution of C_2_ photosynthesis, driven by a fundamental shift in the expression pattern of the GDC subunit GLDP1. In C_3_ plants, GLDP1 is expressed in both MCs and BSCs, but in C_2_ plants, it is predominantly expressed in BSCs, with significantly reduced or silenced expression in MCs. This shift is regulated by cis-regulatory elements and transcription factors. Key cis-elements (e.g., M-box) have been identified as mediators of MC expression, and transcription factor binding sites (e.g., MYC and MYB) within the GLDP1 promoter region collectively modulate GLDP1 expression levels [61]. Transposon insertion plays a critical role in reshaping the evolution of C_2_ photosynthesis. In *Arabidopsis thaliana* (a C_3_ plant), regulatory sequences within the GLDP1 promoter ensure its partial expression in MCs. However, in C_2_ species such as *Moricandia arvensis*, transposon insertion deactivates the cis-elements (e.g., M-box) responsible for maintaining MC expression, thus restricting GLDP1 expression to BSCs. This shift in expression pattern is essential for the glycine shuttling mechanism central to C_2_ photosynthesis. Notably, this transposon-mediated reshaping of GDC expression patterns has occurred independently in several distantly related plant lineages, such as Brassicaceae, Asteraceae, and Poaceae, providing an example of convergent evolution at the molecular level [62]. Metabolomic studies have revealed unique metabolic signatures in C_2_ plants, including distinct patterns of metabolite accumulation (e.g., glycine, serine, and malate), which reflect active carbon and nitrogen shuttling [63]. Additionally, α-ketoglutarate (AKG) has been identified as a critical regulator in the transition from C_2_ to C_4_ photosynthesis by modulating nitrogen metabolism and carbon flux, thereby promoting the evolution of the C_4_ metabolic pathway [64]. Research on the molecular regulatory networks governing C_2_ photosynthesis is still in its early stages. Only approximately 70 C_2_ plant species have been identified (Table 1), and significant gaps remain in understanding the molecular mechanisms that govern traits specific to C_2_ plants, such as leaf anatomical differentiation and gene expression patterns.

## 4. Physiological Ecology Perspectives on the Resilience of C_2_ Photosynthesis

Assimilating and fixing carbon dioxide through photosynthesis is an efficient, economical, and sustainable mechanism for regulating global carbon sink capacity. Photosynthesis not only converts atmospheric carbon dioxide into valuable biomolecules (e.g., proteins, lipids, and carbohydrates) but also captures solar energy and converts it into bioenergy, thereby supporting the proper functioning of global energy and carbon cycles.

### 4.1. Intrinsic Resilience: High-Efficiency Carbon Capture and Energy Conservation

**Figure 2:**
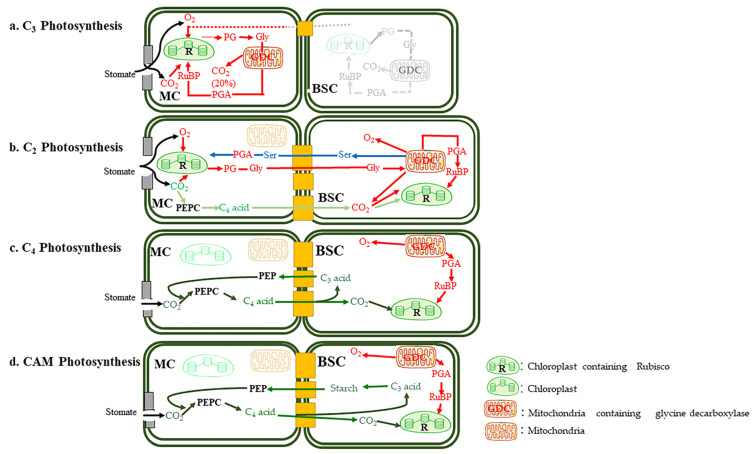
(**a**) C_3_, (**b**) C_2_, (**c**) C_4_ and (**d**) CAM subtype photosynthesis.

Historically, research on carbon fixation and sequestration has primarily focused on C_3_ and C_4_ plants. However, increasing attention is now being directed toward C_2_ photosynthesis. The carbon fixation pathways of C_3_, C_4_, and C_2_ plants are depicted in Figure 2, highlighting key differences in CO_2_ absorption, utilization, and photorespiration.

The oval box indicates the enzymes involved in carbon assimilation and photorespiration. Light gray arrows denote areas of low or negligible metabolic activity, while darker green arrows signify regions of heightened carbon assimilation capacity. Darker orange arrows represent areas with increased photorespiratory activity. Yellow boxes correspond to plasmodesmata. Red arrows illustrate the concurrent processes of photorespiration and carbon assimilation, whereas blue arrows depict the distinct amino acid transport mechanisms in C_2_ plants. Green arrows highlight the carbon assimilation pathways specific to C_2_ and C_4_ plants.

(a) C_3_ photosynthesis: In C_3_ plants, CO_2_ enters through the stomata and is fixed by Rubisco in the chloroplasts of both mesophyll and bundle sheath cells (when chloroplasts are present). The byproducts of photorespiration are recycled within the mitochondria via the glycine shuttle, releasing CO_2_ as a byproduct.

(b) C_2_ photosynthesis: In C_2_ plants, bundle sheath cells exhibit an increased size, and the glycine shuttle is specifically localized within the mitochondria of these cells. The products of photorespiration are subsequently transported back to the mesophyll cells, where they are utilized in photosynthesis.

(c) C_4_ Photosynthesis: In C_4_ plants, Kranz anatomy is present, facilitating the full C_4_ cycle. CO_2_ is initially assimilated in the mesophyll cells and subsequently transferred to the bundle sheath cells, where Rubisco is localized to perform the Calvin–Benson cycle.

(d) CAM Photosynthesis: In CAM plants, CO_2_ is initially assimilated in the mesophyll cells during the night and subsequently transferred to the bundle sheath cells during the day. Within the bundle sheath cells, Rubisco is localized to facilitate the Calvin–Benson cycle.

Both C_2_ and C_3_ plants rely on Rubisco for initial CO_2_ fixation, which competes with O_2_ molecules. However, C_2_ plants exhibit enhanced CO_2_ assimilation capacity due to the re-fixation of photorespiratory CO_2_ in the BSCs, enabling efficient CO_2_ absorption even under low CO_2_ concentrations [60]. Additionally, some C_2_ plants display moderate phosphoenolpyruvate carboxylase (PEPC) activity, further facilitating CO_2_ uptake and utilization [50]. In contrast, C_4_ photosynthesis fixes CO_2_ into organic acids via PEPC, which significantly reduces the proportion of CO_2_ released through stomata, thus offering distinct advantages over both C_2_ and C_3_ plants. However, C_4_ photosynthesis is severely inhibited under low temperatures, diminishing its advantages [65]. Photorespiration, a metabolic pathway that consumes energy and carbon during photosynthesis, varies significantly among different photosynthetic types (refer to Supplementary Materials Table S2 for detailed comparisons of photorespiratory parameters among C_2_, C_3_, and C_4_ plants). In C_3_ plants, photorespiratory carbon loss can exceed 20% of photosynthetically fixed carbon, with most of the released CO_2_ not being re-fixed [60]. In contrast, C_2_ plants optimize photorespiration through a metabolic division of labor between MCs and BSCs, significantly improving the recovery rate of photorespiratory carbon [66]. The markedly lower CO_2_ compensation point of C_2_ plants (compared to C_3_ plants) further attests to the efficiency of their carbon concentration mechanism [60]. For example, in *Flaveria* species, the CO_2_ compensation point of C_2_ species lies between that of C_3_ and C_4_ species, but closer to C_4_ species, reflecting a higher carbon sink efficiency than C_3_ plants [67]. Both empirical measurements and model simulations indicate that, under normal environmental conditions, C_2_ plants generally exhibit stronger carbon capture and assimilation capabilities than closely related C_3_ plants (refer to Supplementary Materials Table S3). While C_4_ plants rely on the C_4_ cycle to increase CO_2_ concentration in BSCs and strongly inhibit photorespiration, this pathway requires additional ATP to drive PEPC-mediated CO_2_ fixation. In contrast, C_2_ photosynthesis leverages the energy generated by photorespiration to concentrate CO_2_, eliminating the need for additional energy input [68]. From an energy efficiency standpoint, C_2_ photosynthesis is more energy-efficient than the C_4_ pathway, conferring a competitive advantage in resource-limited environments. It is also worth noting that the carbon-concentrating efficiency of the C_2_ pathway has a theoretical upper limit, constrained by the rate of photorespiration. By contrast, C_4_ photosynthesis benefits from a more efficient biochemical pump (PEPC), which concentrates CO_2_ in BSCs, minimizing photorespiration and enabling a higher carbon gain—up to 50% more than C_3_ plants under high-temperature and high-light conditions.

### 4.2. Strong Plasticity in Fluctuating Environments

C_2_ photosynthesis exhibits remarkable plasticity, enabling adaptation to fluctuating environmental conditions. This plasticity arises from the synergistic interaction of multiple biological levels, as outlined below: Under stress conditions, C_2_ plants exhibit unique gene expression profiles that distinguish them from C_3_ and C_4_ species, enabling rapid environmental adaptation [13,53]. For instance, certain C_2_ species in families such as Amaranthaceae and Chenopodiaceae upregulate the expression of photosynthetic enzyme genes (e.g., PEPC and NADP-ME) while downregulating specific transporter genes under stress, forming a stress-adapted gene regulatory network that supports biochemical and metabolic adjustments [53]. Building on gene expression regulation, C_2_ plants can upregulate key enzyme activities in response to different stress types, thereby maintaining photosynthetic metabolism. For example, under high-temperature and drought stress, several C_2_ plants significantly enhance GDC activity to ensure the proper functioning of the photorespiratory pump. Under salt stress, *Sedobassia sedoides* increases the activity of core enzymes involved in cyclic electron transport, thereby enhancing cyclic electron flow to supplement ATP production for metabolic processes and improving stress response efficiency [24]. Enzyme activity adjustments optimize metabolic flux distribution and activate synergistic mechanisms across key metabolic pathways. In response to abiotic stresses (e.g., drought and high temperatures), certain C_2_ plants activate the synergistic interaction between photorespiratory carbon recovery and PEPC-assisted carbon fixation through hierarchical regulation of genes and enzymes. This redundancy minimizes the impact of environmental fluctuations on metabolic processes [50]. Moreover, the redistribution of metabolic flux enhances the utilization of nutrients such as nitrogen and sulfur. These three levels of adjustment enable C_2_ plants to maintain higher water use efficiency (WUE) and carbon assimilation under drought and high-temperature stress, as well as adapt to salt stress by enhancing C_4_ traits (e.g., increased PEPC activity) or reducing C_2_ traits, further demonstrating their flexibility in responding to diverse stresses [69,70,71,72,73]. Empirical experiments confirm the carbon sink resilience of C_2_ photosynthesis under various stress conditions, including combined stresses (refer to Supplementary Materials Table S4). Model simulations also indicate that introducing the C_2_ mechanism into C_3_ crops (e.g., rice) results in a stable ~10% increase in carbon sequestration under most conditions, without the potential losses observed in C_4_ plants under high-CO_2_ or low-irradiance environments [18,68]. These findings suggest that C_2_ photosynthesis can maintain stable carbon sinks in fluctuating environments through synergistic molecular-to-physiological mechanisms, offering unique advantages in mitigating global climate change. However, while C_2_ plants are widely distributed, they tend to occupy specific ecological niches and, though advantageous in stress-prone environments, often exhibit lower productivity compared to C_4_ plants in resource-abundant settings.

### 4.3. Advantage Analysis of C_2_ Photosynthesis Compared to C_4_ Photosynthetic Engineering Modifications

Under certain environmental conditions, C_4_ plants require less nitrogen and water than C_3_ plants, resulting in higher photosynthetic productivity [74]. This gives C_4_ plants a stronger carbon sink capacity in grassland and agricultural ecosystems. As global temperatures continue to rise, the advantages of C_4_ plants over C_3_ plants in carbon sink function are expected to increase [75]. Considering this, bioengineering efforts have aimed to transform C_3_ plants into C_4_-functional plants, as exemplified by projects such as the C_4_ Rice Project [76] and the C_4_ transformation of wheat [77]. While these efforts have enhanced photosynthetic carbon assimilation and drought adaptation, they face significant challenges, including structural modifications (e.g., increasing minor vein density and expanding BSC area) and genetic hurdles (e.g., gene silencing, interspecific incompatibility, and multi-gene co-expression). Recent studies suggest that C_2_ photosynthetic modification represents a more feasible alternative to full C_4_ transformation [16,68]. The theoretical and practical advantages of C_2_ modification are as follows: Model simulations indicate that incorporating the C_2_ mechanism into C_3_ crops (e.g., rice) requires fewer structural modifications compared to full C_4_ transformation. For example, studies on the C_2_ plant *Alloteropsis semialata* have shown that C_3_ and C_2_ populations differ only in the number of MCs between leaf veins—C_2_ plants have 3–6 MCs, while C_3_ plants have 5–11 MCs. Notably, vein density does not differ between C_3_ and C_2_ phenotypes of *A. semialata*, whereas C_4_ plants exhibit significantly higher minor vein density [78]. All the biochemical genes required for the C_2_ photosynthetic pathway are already present in C_3_ species. Therefore, reconstructing the glycine shuttling mechanism requires modifying the regulatory and expression patterns of existing genes. In contrast to C_4_ transformation, which involves introducing novel metabolic pathways and structural changes, C_2_ modification necessitates fewer genetic alterations and lower metabolic flux demands, making it a more straightforward approach [68]. Gene editing technologies provide a robust platform for engineering C_2_ crops. Current research employs two main strategies for C_2_ transformation: (1) Targeted knockdown of mesophyll GDC H-subunit (GDCH) expression, such as using artificial microRNA (amiRNA) to knock out GDCH expression in rice MCs [79], simulating the reduced GDC activity in C_2_ plant MCs; and (2) Editing of GDC promoters (e.g., M-box) to preferentially reduce GDC activity in MCs, creating a photorespiration-deficient rice model that mimics C_2_ photosynthesis [80]. Studies have demonstrated that the M-box promoter region, present in C_3_-type *Moricandia* species but absent in C_2_ types, is key to restricting GDC expression to BSC mitochondria in other C_3_ plants, providing new possibilities for C_2_ photosynthetic engineering [16,61]. It should be emphasized that genetic engineering of the C_2_ pathway poses challenges, including potential metabolic perturbations. For example, limiting GDC expression to BSCs may interfere with metabolite partitioning between mesophyll cells and BSCs, potentially impacting nitrogen metabolism and overall plant growth. Furthermore, the release of ammonia (NH_3_) during glycine decarboxylation could cause cellular toxicity if reassimilation pathways are impaired.

### 4.4. Ecological Perspectives on the Resilient Carbon Sink of C_2_ Photosynthesis

C_2_ photosynthesis has evolved through convergent evolution in response to declining atmospheric CO_2_ concentrations, high temperatures, and drought conditions [27,70,81]. Studies suggest that reduced precipitation and increasing temperatures are linked to transitions from C_3_ to C_2_ photosynthesis, and from C_2_ to incipient C_4_ photosynthesis. While drought, rather than heat, may primarily drive the evolution of C_4_ photosynthesis, this varies across evolutionary stages, with the initial transition to C_2_ photosynthesis requiring increased water availability [70]. Compared to C_3_ plants, C_2_ plants exhibit higher carbon sink stability and lower photorespiratory losses under high-temperature and high-irradiance conditions [16,68]. Furthermore, C_2_ plants do not require the complex Kranz anatomy or high energy input necessary for C_4_ photosynthesis, making them more cost-effective in moderately stressed or resource-limited environments [68]. These traits enable C_2_ plants to serve as significant carbon sinks in specific ecological niches. Complementary Niche Differentiation: In mixed communities of cotton (C_3_) and sorghum (C_4_), CO_2_ enrichment increases total biomass and leaf area, mitigating the competitive suppression of C_3_ plants by C_4_ plants [82]. This suggests that under future elevated CO_2_ conditions, rational species assemblage within communities can offset the negative impacts of interspecific competition. Although data on the competitive performance of C_2_ plants in community contexts are limited, niche analysis reveals minimal overlap among C_2_, C_3_, and C_4_ species. For example, in *Diplotaxis*, the C_2_-type *D. tenuifolia*, C_3_-type *D. muralis*, and C_4_-type *Cleome gynandra* exhibit distinct light saturation point gradients, enabling effective resource utilization across both temporal and spatial niche dimensions. Niche analysis indicates that C_2_ species typically occupy transitional environments characterized by seasonal rainfall and warm temperatures, bridging the ecological niches of C_3_ and C_4_ plants [25]. This distribution often overlaps with savanna habitats, which contribute significantly more to global carbon sinks than temperate forests [83]. Given their evolved ability to adapt to fluctuating precipitation patterns, C_2_ plants are well-suited to serve as “carbon sink buffers” amid climate change. Notably, in regions impacted by El Niño and La Niña climate phenomena, C_3_ and C_4_ plants are highly vulnerable to severe damage, while C_2_ plants exhibit relatively stronger resilience. Thus, strategically introducing C_2_ plants to such areas could serve as a cost-effective approach to reducing ecosystem risks and enhancing carbon sink stability. Ecological restoration of marginal lands, such as arid, salinized, and barren soils, presents a global challenge. C_2_ plants demonstrate superior carbon fixation capabilities in these environments compared to C_3_ plants, making them promising candidates for ecological restoration projects [25]. Their advantages include high water use efficiency (WUE), efficient carbon assimilation under low CO_2_ concentrations, and strong adaptability to barren soils, all of which are achieved through optimized nitrogen metabolism [33,63]. Case studies from arid and semi-arid regions further confirm that C_2_ plants outperform C_3_ and C_4_ plants in terms of both adaptability and carbon sink functionality [25].

## 5. Conclusions 

This review systematically examines the mechanistic basis, carbon sink resilience, and potential applications of C_2_ photosynthesis, drawing the following key conclusions: C_2_ photosynthesis enhances carbon sink resilience through its photorespiratory carbon pump mechanism, which facilitates CO_2_ concentration in the BSCs, significantly reduces the CO_2_ compensation point, and maintains exceptional carbon sink stability under low CO_2_ levels, high temperatures, and drought conditions. This adaptation exemplifies a sophisticated physiological response, underscoring the evolutionary ingenuity of biological systems [60,66]. C_2_ photosynthesis demonstrates remarkable convergent evolution across different plant lineages. This photosynthetic pathway plays a vital physiological and ecological role in mitigating the impacts of global climate change and enhancing the carbon sink resilience of the biosphere. The widespread occurrence of C_2_ photosynthesis across diverse plant families highlights its adaptive response to environmental stressors such as reduced CO_2_ availability and elevated temperatures [27,70,81]. Compared to C_3_ and C_4_ plants, C_2_ plants exhibit superior carbon sink resilience. Furthermore, C_2_ modification presents fewer technical challenges than C_4_ engineering, making it a more technically feasible strategy for enhancing carbon sequestration in both crops and ecosystems. These advantages position C_2_ photosynthesis as a promising tool for ecological applications, particularly in the management of marginal lands, where factors such as drought and poor soil quality limit the growth of traditional crops. Given the ongoing challenges posed by global climate change, harnessing the resilient carbon sink function of C_2_ plants presents a practical approach to achieving long-term stable carbon sinks [16,68].

However, despite these advantages, several critical research gaps remain in the study of C_2_ photosynthesis. Research into the physiological mechanisms underlying C_2_ photosynthesis is still in its infancy. Further investigation is urgently required in areas such as the regulation of small RNAs, epigenetic modifications, and the precise regulation of gene expression at the genomic level. Such studies are essential to fully understand how C_2_ photosynthesis operates and how it can be optimized for crop species [53,61]. Moreover, currently available biotechnologies, such as gene editing, high-throughput sequencing, and single-cell sequencing, should be promptly applied to research on the distinct characteristics of the C_2_ photosynthetic pathway. Although C_2_ modification is technically less complex than C_4_ engineering, challenges persist in optimizing the cost-effectiveness, environmental adaptability, metabolic compatibility, and genetic stability of the engineered traits. Overcoming these challenges is crucial for advancing the practical application of C_2_ photosynthesis in agriculture [68,79]. Ecological research pertaining to C_2_ plants remains relatively underdeveloped. Further studies are needed to explore the competitive interactions of C_2_ plants at the individual, community, and ecosystem levels, including understanding both aboveground and belowground interactions and the role of C_2_ plants in modifying soil microbial communities. These studies will be pivotal in predicting the ecological impacts of introducing C_2_ plants into various environments and optimizing their carbon sink potential [25,84].

In summary, C_2_ photosynthesis holds considerable promise as a resilient carbon sink mechanism that could play a significant role in mitigating the effects of global climate change. As research advances, the full potential of C_2_ photosynthesis for both ecological restoration and agricultural enhancement is likely to be realized.

## Figures and Tables

**Table 1:** C_2_ species by family and lineage.

Family	Lineage	C_2_ Species	References
***Monocots***			
Poaceae	Alloteropsis	*Alloteropsis semialata*	[16]
	Homolepis	*Homolepis aturensis*, *H. isocalycia*, *H. longispicula*	[16,17]
	Neurachne	*Neurachne minor*	[16,18,19]
	Steinchisma	*Steinchisma cuprea, S. decipiens*, *S. hians*, *S. spathellosa*, *S. exiguiflora*, *S. spathellosum*, *S. stenophylla*	[16,17,20,21]
***Eudicots***			
Acanthaceae	Blepharis	*Blepharis acuminate, B. diversispina*, *B. espinosa*, *B. gigantea*, *B. natalensis*, *B. nolimetangere, B. sinuate, B. pruinose*, *B. subvolubilis*	[16]
Amaranthaceae	Alternanthera	*Alternanthera cruci*, *A. ficoidea*, *A. tenella*	[16,20]
	Salsola	*Salsola arbusculiformis*, *S. divaricate*, *S. deschaseauxiana*, *S. gymnomaschala*, *S. verticillate*, *S. laricifolia*	[21,22,23,24]
	Sedobassia	*Sedobassia sedoides*	[16,25]
Asteraceae	Flaveria	*Flaveria angustifolia*, *F. anomala, F. chloraefolia*, *F. floridana, F. linearis, F. oppositifolia*, *F. pubescens*, *F*. *ramosissima*, *F. sonorensis*	[16,20,26,27,28,29,30]
	Parthenium	*Parthenium hysterophorus*	[16,31]
Boraginaceae	Heliotropium	*Heliotropium convolvulaceum*, *H. greggii*, *H. racemosum, H. lagoense*	[16,32,33,34]
Brassicaceae	Brassica	*Brassica gravinae*	[16]
	Diplotaxis	*Diplotaxis erucoides*, *D. tenuifolia*, *D. muralis*	[16,35]
	Moricandia	*Moricandia arvensis*, *M. nitens*, *M. suffruticosa*, *M. sinaica*, *M. spinosa*	[16,35,36]
Cleomaceae	Cleome	*Cleome paradoxa*	[16,37,38]
Euphorbiaceae	Euphorbia	*Euphobia acuta, E. johnstonii*, *E.racemosa*	[39,40,41]
	Hypertelis	*Hypertelis spergulacea*	[16]
Molluginaceae	Paramollugo	*Paramollugo nudicaulis*	[41]
	Mollugo	*Mollugo verticillata*	[16,26,41]
Nyctaginaceae	*Bougainvillea*	*Bouganvillea cv. Mary Palmer*	[42]
Portulacaceae	Portulaca	*Portulaca cryptopetala*, *P. hirsutissima, P. mucronate*, *P. amillis*, *P. biloba*, *P. elatior, P. smallis*	[16,43,44]
Scrophulariaceae	Anticharis	*Anticharis ebracteate, A. juncea*	[16,45]
Zygophyllaceae	Tribulus	*Tribulus cristatus*, *T. astrocarpus*	[46]

## Data Availability

All data generated or analyzed during this study are included in this published article.

## Supplementary Materials

The following supporting information can be downloaded at: https://www.mdpi.com/article/10.3390/biology15010005/s1, Table S1: Comparative Analysis of leaf anatomical feature Across C_3_, C_2_, and C_4_ Species; Table S2: Comparative Analysis of Photorespiration Traits Across C_3_, C_2_, and C_4_ Species; Table S3: Comparative Analysis of Photosynthetic Traits Across C_3_, C_2_, and C_4_ Species under normal condition; Table S4: Comparative Analysis of Photosynthetic Traits Across C_3_, C_2_, and C_4_ Species under stress condition [85,86,87,88,89,90,91,92,93,94].
