# Supplementary material for: C2 Resilient Photosynthesis: A Practical Option for Long-Term Stable Carbon Sinks?
Source: Biology (Basel). 2025 Dec 19;15(1):5. doi: 10.3390/biology15010005 (PMC12784988; doi:10.3390/biology15010005)
Supplement: Supplementary file 1 [file biology-15-00005-s001.zip › biology-4026804-supplementary.pdf]

Table S1. Comparative Analysis of leaf anatomical feature Across C_3, C_2, and C_4 Species

Families and lineage	Key results of leaf anatomical feature
1. Amaranthaceae: *Alternanthera*[20], *Salsoleae*[23,85], *Alloteropsis*[78]<br>2. Brassicaceae: *Diplotaxis*[86]<br>3. Boraginaceae: *Heliotropium*[33,34], *Euploca*[87]<br>4. Asteraceae: *Flaveria*[60,69,71]<br>5. Molluginaceae: *Mollugo*[88]<br>6. Poaceae: *Homolepis*[15], *Neurachne*[89,90]<br>7. Modeling studies: *Oryza sativa*[68], *Flaveria*[91]	1. Proportion of bundle sheath cells (BSC) in number and area: $C_3 < C_2 < C_4$<br>2. BSC/MC size ratio[1], BSC organelle concentration (chloroplasts/mitochondria): $C_3 < C_2 < C_4$<br>3. BSC/MC organelle concentration ratio, proportion of chloroplasts in BSC, % M+P[2]: $C_3 < C_2 < C_4$<br>4. Vein density of some species: $C_3 < C_2 < C_4$
1. Cleomaceae: *Cleome*[37]<br>2. Amaranthaceae: *Atriplex*[92], *Chenopodium*[93]<br>3. Zygophyllaceae: *Tribulus*[46]	1. *Cleome*: BSC/MC size ratio, BS:M area ratio[3]: $C_3 < C_4 < C_2$<br>2. *Atriplex*: BSC chloroplast size: $C_2 \approx C_3 < C_4$<br>3. *Chenopodium*: BSC number/area proportion, BSC/MC size ratio: $C_2 \approx C_3$; BSC organelle concentration: $C_3 < C_2$<br>4. *Tribulus*: BSC mitochondrial concentration: $C_3 < C_4 < C_2$; BSC area proportion, BSC/MC size ratio: $C_2 < C_3 < C_4$
Brassicaceae: *Moricandia*[94]	1. BSC number/area proportion, BSC/MC size ratio, BSC organelle concentration: $C_3 < C_2$<br>2. Proportion of chloroplasts in BSC, % M+P: $C_3 < C_2$
Poaceae: *Steinchisma*[15]	M:BS tissue ratio [4]$C_4 < C_2 \approx C_3$

[1]BSC/MC size ratio: Bundle sheath cell/mesophyll cell size ratio;

[2]% M+P in BSC: Percentage of mitochondria + peroxisomes located in bundle sheath cells.

[3]BS:M area ratio: The ratio of the tissue area of bundle sheath (BS) cells to mesophyll (M) cells;

[4]M:BS tissue ratio: The ratio of the tissue area of mesophyll (M) cells to bundle sheath (BS) cells

Table S2. Comparative Analysis of Photorespiration Traits Across C_3, C_2, and C_4 Species

Families: Genera	Key Photorespiration Traits Under Normal Conditions	Key Photorespiration Traits Under Stress Conditions
1. Amaranthaceae: *Alternanthera*[1]; *Salsoleae*[23]; *Sedobassia*[95]; *Alloteropsis*[78]<br>2. Brassicaceae: *Diplotaxis*[86]<br>3. Boraginaceae: *Heliotropium*[33,34]; *Euploca*[87]<br>4. Cleomaceae: *Cleome*[37]<br>5. Asteraceae: *Flaveria*[60,69,71]<br>6. Molluginaceae: *Mollugo*[88]<br>7. Poaceae: *Homolepis*[15]; *Neurachne*[89,90]<br>8. Modeling study: *Oryza sativa*[68]	1. Pr[1], O_2 inhibition rate, glycolate content, Gly/Ser ratio: $C_4 < C_2 < C_3$<br>2. CE[2], CO_2 refixation rate, D[3]: $C_3 < C_2 < C_4$<br>3. Γ[4](21% O_2): $C_4 < C_2 < C_3$	1. Under drought, salt stress, and low CO_2, the above indicators still maintain $C_3 < C_2 < C_4$<br>2. *Flaveria* (drought): ΔPr, ΔO_2 inhibition rate: $C_4 < C_2 < C_3$; ΔCE: $C_3 < C_2 < C_4$
1. Amaranthaceae: *Atriplex*[92], *Chenopodium*[93]<br>2. Brassicaceae: *Moricandia*[72,94]<br>3. Modeling study: *Flaveria*[91] (partial)	1. *Atriplex*: CE: $C_2 < C_3 < C_4$<br>2. *Moricandia* (compared only with C_3): Pr, O_2 inhibition rate: $C_2 < C_3$; CE, D: $C_2 > C_3$<br>3. *Chenopodium* (compared only with C_3): Pr: $C_2 < C_3$; CO_2 refixation rate: $C_2 > C_3$<br>4. Modeled *Flaveria*: CE: $C_2 < C_3 < C_4$	1. *Moricandia* (drought): ΔPr, ΔO_2 inhibition rate: $C_2 < C_3$; ΔD: $C_2 > C_3$<br>2. *Chenopodium* (low nitrogen): Pr: $C_2 < C_3$; CE: C_2 (low nitrogen) < C_2 (standard nitrogen) = C_3<br>3. Modeled *Flaveria* (low CO_2): CE still maintains $C_2 < C_3 < C_4$
Zygophyllaceae: *Tribulus*[46]	1. Pr, O_2 inhibition rate, C[5]* (CO_2 compensation point without dark respiration): $C_4 < C_2 < C_3$<br>2. CE, CO_2 refixation rate: $C_3 < C_2 < C_4$	At 42℃ high temperature, all indicators are consistent with normal conditions
Poaceae: *Steinchisma*[15]; *Neurachne*[89,90]	1. Pr, O_2 inhibition rate: $C_4 < C_2 < C_3$; CO_2 refixation rate: $C_3 < C_2 < C_4$<br>2. CE, A_n: $C_2 \approx C_3 < C_4$	No data

[1]Pr: Photorespiration rate;

[2]CE: Carboxylation efficiency;

[3]D: CO_2 reassimilation coefficient;

[4]Γ: CO_2 compensation point;

[5]C*: CO_2 compensation point without day respiration.

Species under normal condition.

Families/Genera	Gas Exchange Parameters	Enzyme’s activity	Resource Use Efficiency
1. Amaranthaceae: *Alternanthera*[20]; *Salsoleae*[23,85], *Sedobassia*[95]; *Alloteropsis*[78]<br>2. Brassicaceae: *Diplotaxis*[86]<br>3. Boraginaceae: *Heliotropium*[33,34]; *Euploca*[87]<br>4. Cleomaceae: *Cleome*[37]<br>5. Asteraceae: *Flaveria*[60,69,71]<br>6. Molluginaceae: *Mollugo*[88]<br>7. Modeling Study: *Oryza sativa*[68]	1. Γ, A_n, C_i: $C_3 < C_2 < C_4$;<br>2. g_s (partial data): $C_4 < C_2 < C_3$	1. PEP, PPDK, NAD-ME, NADP-ME: $C_3 < C_2 < C_4$;<br>2. Rubisco: $C_4 < C_2 \approx C_3$	WUE: $C_3 < C_2 < C_4$; NUE of some species: $C_3 < C_2 < C_4$
1. Amaranthaceae: *Atriplex*[92], *Chenopodium*[93]<br>2. Brassicaceae: *Moricandia*[72,94]<br>3. Zygophyllaceae: *Tribulus*[46]<br>4. Modeling Study: *Flaveria*[91] (partial data)	1. *Atriplex*: A_n, WUE: $C_2 \approx C_3 < C_4$; CE: $C_2 < C_3 < C_4$;<br>2. *Moricandia/Chenopodium* (compared only with C_3): Γ: $C_2 < C_3$; A_n, C_i, g_s: $C_2 \approx C_3$<br>3. *Tribulus*: Γ, C*, A_n: $C_3 < C_2 < C_4$; g_s: $C_2 \approx C_3 > C_4$<br>4. Modeled *Flaveria*: CE: $C_2 < C_3 < C_4$; WUE: $C_2 \approx C_3 < C_4$	1.*Atriplex*: PEPC, PPDK: $C_3 < C_2 < C_4$; Rubisco: $C_4 < C_2 < C_3$;<br>2. *Moricandia/Chenopodium*: Rubisco: $C_2 \approx C_3$; PEPC: $C_2 \approx C_3$<br>3. *Tribulus*: PEPC, NADP-ME: $C_2 \approx C_3 < C_4$; AlaAT, AspAT: $C_3 < C_2 < C_4$<br>4. Modeled *Flaveria*: no specific deviations	1. Atriplex: NUE: $C_2 < C_3 < C_4$<br>2. Moricandia/Chenopodium: WUE, NUE: $C_2 \approx C_3$<br>3. Tribulus: No specific deviations
Poaceae: *Steinchisma*[15], *Homolepis*[15], *Neurachne*[89,90]	1. Γ, C*: $C_3 < C_2 < C_4$; A_n, CE: $C_2 \approx C_3 < C_4$<br>2. g_s: $C_2 \approx C_3 > C_4$	1. *Steinchisma/Homolepis*: PEPC: $C_2 \approx C_3 < C_4$<br>2. *Neurachne*: PEPC, PPDK: $C_3 < C_2 < C_4$	WUE, NUE: $C_2 \approx C_3 < C_4$

[1]CE: Carboxylation efficiency;

[2]C*: CO_2 compensation point without day respiration;

[3]PEPC: Phosphoenolpyruvate carboxylase;

[4]PPDK: Pyruvate orthophosphate dikinase;

[5]NAD-ME: NAD-Malic Enzyme;

[6]NADP-ME: NADP-Malic Enzyme;

[7]Rubisco: Ribulose-1,5-Bisphosphate carboxylase/oxygenase;

[8]AlaAT: Alanine Aminotransferase;

[9]AspAT: Aspartate Aminotransferase.

[10]Water Use Efficiency

[11]Nitrogen Use Efficiency

Table S4. Comparative Analysis of Photosynthetic Traits Across C_3, C_2, and C_4 Species under stress condition.

[1]Pr: Photorespiratory rate;

Families:Genera	Stress Type	Gas Exchange Parameters	Enzymes activity	Resource Use Efficiency
1. Amaranthaceae: *Salsoleae*[23], *Sedobassia*[95], *Alloteropsis*[78] 2. Asteraceae: *Flaveria*[71] 3. Zygophyllaceae: *Tribulus*[46]	1. Drought (PEG), Salt stress (2% NaCl) 2. Low CO_2 (180-280 μmol mol^{-1})	1. Γ, A_n, C_i, CE: $C_3 < C_2 < C_4$ 2. *Flaveria* (under drought): ΔA_n, Δg_s: $C_4 < C_2 < C_3$	1. *Salsoleae*: PEPC, NADP-ME: $C_3 < C_2 < C_4$; PEP-CK: $C_3 < C_4 < C_2$ 2. *Flaveria* (under drought): ΔPEPC: $C_3 < C_2 < C_4$; ΔRubisco: $C_4 < C_2 < C_3$	WUE: $C_3 < C_2 < C_4$
1. Brassicaceae: *Moricandia*[72,94] 2. Amaranthaceae: *Chenopodium*[93]	1. Drought (Soil water potential: -0.25~-1.8 MPa) 2. Low nitrogen (0.05 g N / pot)	1. *Moricandia* (under drought): ΔA_n, Δg_s, ΔPr^1: $C_2 < C_3$ (smaller variation range) 2. *Chenopodium* (under low nitrogen): A_n, CE: C_2 (low nitrogen) < C_2 (standard nitrogen) = C_3; Γ: $C_2 < C_3$	1. *Chenopodium* (under low nitrogen): Rubisco: $C_2 \approx C_3$ 2. *Moricandia*: No data available	1. *Moricandia*: WUE: $C_2 \approx C_3$ 2. *Chenopodium*: WUE, NUE: $C_2 \approx C_3$
Modeling study: *Oryza sativa*[68]	1. Low light (<300 μmol $m^{-2}s^{-1}$) 2. Low temperature (<20℃) 3. Low CO_2 (180 μmol mol^{-1})	1. Low light/low temperature: A_n, WUE: $C_4 < C_3 < C_2$ 2. Low CO_2: A_n, WUE: $C_3 < C_2 < C_4$ 3. All stresses: Γ, CE, g_s: $C_3 < C_2 < C_4$	PEPC (under all stresses): $C_2 \approx C_3 < C_4$	1. Low light/low temperature: WUE: $C_3 < C_4 < C_2$ 2. Low CO_2: WUE: $C_3 < C_2 < C_4$
Zygophyllaceae: *Tribulus*[46]	High temperature (42℃)	Γ, C*, A_n, CE: $C_3 < C_2 < C_4$ (consistent with normal conditions)	PEPC, NADP-ME: $C_2 \approx C_3 < C_4$ (consistent with normal conditions)	No significant changes
